# Left Atrial Cardiomyopathy – A Challenging Diagnosis

**DOI:** 10.3389/fcvm.2022.942385

**Published:** 2022-06-30

**Authors:** Fabienne Kreimer, Michael Gotzmann

**Affiliations:** University Hospital St. Josef-Hospital Bochum, Cardiology and Rhythmology, Ruhr University Bochum, Bochum, Germany

**Keywords:** atrial cardiomyopathy, diagnosis, atrial fibrillation, embolic stroke of undetermined source, diagnostic algorithm

## Abstract

Left atrial cardiomyopathy (LACM) has been an ongoing focus of research for several years. There is evidence that LACM is responsible for atrial fibrillation and embolic strokes of undetermined sources. Therefore, the correct diagnosis of LACM is of clinical importance. Various techniques, including electrocardiography, echocardiography, cardiac magnetic resonance imaging, computed tomography, electroanatomic mapping, genetic testing, and biomarkers, can both identify and quantify structural, mechanical as well as electrical dysfunction in the atria. However, the question arises whether these techniques can reliably diagnose LACM. Because of its heterogeneity, clinical diagnosis is challenging. To date, there are no recommendations for standardized diagnosis of suspected LACM. However, standardization could help to classify LACM more precisely and derive therapeutic directions to improve individual patient management. In addition, uniform diagnostic criteria for LACM could be important for future studies. Combining several parameters and relating them seems beneficial to approach the diagnosis of LACM. This review provides an overview of the current evidence regarding the diagnosis of LACM, in which several potential parameters are discussed and, consequently, a proposal for a diagnostic algorithm is presented.

## Highlights

- Left atrial cardiomyopathy (LACM) is a common disease associated with histopathological detectable structural and/or electrical changes in the myocardium of the left atrium.
- The clinical significance of LACM arises from its association with atrial fibrillation and embolic strokes of undetermined sources. Early detection of LACM may hence be important for the prevention of stroke.
- Various methods (including electrocardiography, echocardiography, cardiac magnetic resonance imaging, computed tomography, electroanatomic mapping, genetic testing, and biomarkers) have been investigated to establish the diagnosis of LACM. To date, however, there are no universally accepted recommendations.
- The present review presents the current evidence of the different methods and proposes an algorithm that could be used to diagnose LACM at present.
- The current work could help to make future research comparable and to unify diagnostic criteria of LACM.

## Introduction

Cardiomyopathies can affect all parts of the heart to some extent, although there are types that predominantly involve one specific chamber (1). The idea of an left atrial cardiomyopathy (LACM) is not entirely new, but has recently become more significant and important (1, 2). Patients with lone atrial fibrillation (AF) and patients with embolic stroke of undetermined source (ESUS) are of particular interest because LACM is often suspected as an underlying disorder. Previous genetic studies identified genes that were associated with AF as well as electrical and structural left atrial (LA) remodeling, e.g., MYL4, Lamin-A, ETV1, Scn5a, Gja5, ErbB4, Tgfbr1/2, Igf1, TTN, numerous collagen genes (3–8). However, this probably only applies to a very small proportion of all patients with LACM. A detailed family history provides important and decisive clues in which patients genetic testing might be worthwhile.

### Definition of Left Atrial Cardiomyopathy

In 2016, a position paper was published to define, characterize, and derive clinical implications of LACM (9). The expert group defined LACM as “any complex of structural, architectural, contractile or electrophysiological changes affecting the atria with the potential to produce clinically relevant manifestations” (9). The definition is broad and not disease-specific, as both risk factors and comorbidities may implicate and contribute to atrial changes (9). Therefore, a classification was introduced to classify LACM histopathological. Accordingly, LACM is divided into four categories: primarily cardiomyocyte-dependent (class I), primarily fibroblast-dependent (class II), mixed cardiomyocyte-fibroblast-dependent (class III) and primarily non-collagen deposits (class IV). Of course, mixed phenotypes are often present, and since this is a dynamic process, class variation over time is possible (9).

However, the heterogeneity of LACM causes difficulties not only in classification, but particularly in diagnosis. Several techniques have been described to diagnose LACM, e.g., electrocardiography (ECG), echocardiography, cardiac magnetic resonance imaging (MRI), computed tomography (CT), electroanatomic mapping, biomarkers, and genetic testing (9–12). Another complicating factor is that there are a variety of parameters that define LACM in different studies. To date, there is no randomized controlled trial that addresses LACM. Other studies that have attempted to identify parameters for the diagnosis of LACM so far are also extremely heterogeneous and thus difficult to compare. Most studies as well as the consensus paper focused on patients with AF or patients who have suffered stroke. The derived parameters should thus be considered cautiously, as a direct transferability to LACM may be questionable. However, combining the parameters and relating them might help to best approach the diagnosis of LACM.

The aim of this review is therefore to provide an overview of the current diagnostic options for an LACM, to highlight interrelationships of the different methodologies, and to discuss the clinical value.

## Clinical Impact

### Left Atrial Cardiomyopathy and Atrial Fibrillation

Left atrial cardiomyopathy and atrial fibrillation are closely related, but there is a controversy on whether AF is merely a marker of LACM. On the other hand, AF leads to AF and thus most likely promotes LACM, as AF *per se* can also cause atrial remodeling due to changes in ion channels and the development of atrial fibrosis (9). This mechanism conversely leads again to stabilization of AF and progression from lower to higher AF burden. Whereas very brief AF episodes do not alter the degree of fibrosis, longer lasting AF episodes may cause AF-induced LACM (9). Moreover, the presence of subclinical AF, particularly a higher burden, is significantly correlated with an increased thromboembolic risk (13). Therefore, the presumption exists that subclinical AF may be an early manifestation of LACM with increased risk of stroke and not the underlying cause of thromboembolic events (13).

“Lone” AF is also discussed as a possible marker of an existing LACM. “Lone” AF is diagnosed when there is no underlying explanation and other facilitating comorbidities (9). The risk of thromboembolism is lower, with a cumulative 15-year stroke risk of 1–2%, but it increases with the number of cardiovascular risk factors, e.g., age, male sex (9). In patients with “lone” AF, structural atrial remodeling, conduction disturbances, and morphological and inflammatory changes of atrial cardiomyocytes were described (9).

### Left Atrial Cardiomyopathy and Embolic Stroke of Undetermined Source

Ischemic strokes are one of the most common causes of cardiovascular morbidity and mortality. A significant proportion of patients with ischemic stroke are suspected of embolic stroke without a specific embolic source being found (ESUS) (14). The clinical significance of ESUS results from the frequency of the disease and the fact that the recurrence risk for a new stroke is 4–5% per year despite antiplatelet agents (15).

A clinically significant therapeutic implication of this concept was that, because of the embolic genesis, oral anticoagulation rather than antiplatelet therapy might be beneficial for this group of patients.

By contrast, neither the NAVIGATE-ESUS trial comparing rivaroxaban with acetylsalicylic acid nor the RESPECT-ESUS trial with dabigatran demonstrated a statistically significant reduction in ischemic stroke or ischemic MRI lesions (16, 17). Similarly, the results of the ATTICUS trial, which were recently presented at the European Stroke Organisation Conference 2022 but not yet published, demonstrated that apixaban was not superior to acetylsalicylic acid in patients with ESUS and risk factors for cardiac thromboembolism (18).

However, a subgroup analysis of the NAVIGATE-ESUS trial demonstrated that patients with moderate and severe LA dilatation had a significant benefit from therapy with rivaroxaban (19). The results suggest that a proportion of patients with ESUS have LACM, which increases cardioembolic risk.

The randomized-controlled ARCADIA trial will evaluate whether apixaban is superior to aspirin for prevention of recurrent stroke in patients with ESUS and LACM (20). According to the investigators, an LACM is defined by the presence of at least one of the following criteria: P-wave terminal force in lead V1 (PTFV1) > 5,000 μV*ms, Serum NT-proBNP > 250 pg/mL, LA diameter index ≥ 3 cm/m^2^. Findings from the ARCADIA trial would have implications for secondary stroke prevention as well as primary prevention of LACM (20).

## Definition of Left Atrial Cardiomyopathy in Different Studies

**FIGURE 1:**
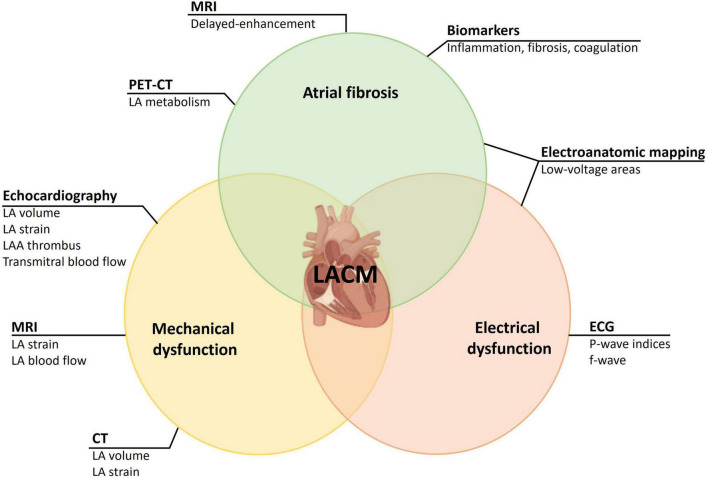
Illustration of LACM as a composite entity of atrial fibrosis, mechanical dysfunction, and electrical dysfunction. Assessment of entities is achieved through different methods. CT, computed tomography; ECG, electrocardiology; f-wave, fibrillatory wave; LA, left atrial/let atrium; LAA, left atrial appendage; LACM, left atrial cardiomyopathy; MRI, magnetic resonance imaging; PET-CT, cardiac positron emission tomography – computed tomography.

Left atrial cardiomyopathy is both a clinical and histological diagnosis (9). The relationship between LACM and AF on the one hand and LACM and ESUS on the other suggests that correct diagnosis of LACM is of clinical benefit. As mentioned above, there is no uniform definition of LACM to date. Several studies have characterized the disease differently and used various methods:

For a correct diagnosis of LACM, histological examination of the atrial myocardium is required. Due to the invasive nature of a LA biopsy, this is an option in very few patients (for example, in mitral valve surgery). For an insight into the histology of the LA, we are thus dependent on methods that can detect fibrosis or scars. For this purpose, cardiac MRI, electroanatomic mapping in electrophysiologic studies, and PET-CT can be used. Further methods are dealing with the effects of an LACM on mechanical or electrical function. To detect mechanical dysfunction due to LACM, echocardiography may be considered in addition to CT and MRI. Furthermore, ECG parameters are suitable for the detection of electrical dysfunction, which in LACM may result from both functional and morphological changes in the LA (Figure 1). To what extent laboratory parameters are suitable to establish or enhance the likelihood of the diagnosis of LACM remains unclear. However, some laboratory parameters seem to be suitable to identify pathophysiological correlations (for example, LACM with immunological and inflammatory functions).

In conclusion, Table 1 summarizes the different variables that have been used as markers of LACM in different studies.

**TABLE 1:** Overview of different methods and variables for the assessment of LACM, and their associations to clinical outcomes and abnormal parameters.

Method	Parameter	Associations	References
Electrocardiography	f-wave	Abnormal echocardiographic parameters, SEC and thrombus in LA appendage, AF burden, age	(21–23)
	PTFV1	Ischemic stroke, AF, BMI, age, abnormal echocardiographic parameters	(24–37, 39)
	P-wave area	Ischemic stroke	(34)
	P-wave duration	Ischemic stroke, BMI, age, blood pressure	(34, 39)
	Advanced interatrial block	AF, Ischemic stroke	(25, 35, 38)
	Amplified P-wave duration	Abnormal echocardiographic parameters, LA appendage thrombus, major adverse cardiovascular events, AF, Recurrence of AF after ablation, low-voltage areas in electroanatomic mapping	(40–43)
	Artificial intelligence probability	Structural heart disease, abnormal echocardiographic parameters, AF, mortality	(45)
	Atrial premature complexes	Abnormal echocardiographic parameters	(46)
Transthoracic echocardiography	LA diameter	AF, ischemic stroke, major adverse cardiovascular events	(48–50)
	LA volume index	Major adverse cardiovascular events, AF, AF burden	(51–56)
	LA emptying fractions	AF, low-voltage areas in electroanatomic mapping, recurrence of AF after ablation	(43, 51, 57)
Doppler echocardiography	E-wave velocities	AF burden	(51, 52)
	A-wave velocities	AF	(51, 59)
	PA-TDI duration	AF, AF recurrence, thromboembolic events	(60)
3-D and 4-D echocardiography	LA volume index	Abnormal delayed-enhancement in MRI	(56)
	LA emptying fractions	Abnormal delayed-enhancement in MRI	(56)
	LA strain	Abnormal delayed-enhancement in MRI	(56)
Speckle-tracking echocardiography	LA strain	AF, AF burden, thromboembolic events, low-voltage areas in electroanatomic mapping, AF recurrence after ablation	(43, 57, 61–68)
	LA mechanical dispersion	AF, low-voltage areas in electroanatomic mapping	(64, 69)
Transesophageal echocardiography	LA appendage thrombus	Abnormal delayed-enhancement in MRI, LA appendage flow dynamics	(70, 71)
	SEC	Abnormal delayed-enhancement in MRI, LA appendage flow dynamics	(70, 71)
Cardiac MRI	LA strain	AF	(72–74)
Delayed- enhancement MRI	Delayed-enhancement in LA wall	AF, low-voltage areas in electroanatomic mapping, abnormal echocardiographic parameters, CHA2DS2-VASc score, ischemic stroke, major adverse cardiovascular events, AF recurrence after ablation	(75–86)
4-D flow MRI	LA blood flow velocities	AF burden, CHA2DS2-VASc score, age, abnormal echocardiographic parameters	(88–91)
CT	LA volume index	AF recurrence after ablation, reproducible in speckle-tracking echocardiography	(92, 93)
	LA strain	Reproducible in speckle-tracking echocardiography	(94–96)
	Image attenuation ratio	Low-voltage areas in electroanatomic mapping	(97)
PET-CT	18F-fluorodeoxyglucose activity	Ischemic stroke, AF, AF burden	(98, 99)
Electroanatomic mapping	Low-voltage areas	AF, CHA2DS2-VASc score, ischemic stroke, silent cerebral ischemia in MRI, AF recurrence after ablation, CRP, abnormal echocardiographic parameters	(100–110)
Biomarkers	Inflammation markers	AF, AF burden, AF recurrence after ablation, low-voltage areas in electroanatomic mapping, abnormal echocardiographic parameters	(112–121)
	Fibrosis markers	AF, AF recurrence after ablation, abnormal echocardiographic parameters, ischemic stroke	(105, 121–125)
	N-terminal pro-B-type natriuretic peptide	AF, abnormal echocardiographic parameters,	(114, 125–129)
	N-terminal pro-A-type natriuretic peptide	Low-voltage areas in electroanatomic mapping	(130)
	Aldosterone	AF	(131)
	Immunothrombosis markers	AF, major adverse cardiovascular events	(134–140)

*AF, atrial fibrillation; BMI, body mass index; CRP, C-reactive protein; CT, computed tomography; f-wave, fibrillatory wave; LA, left atrial/left atrium; MRI, magnetic resonance imaging; PET-CT, cardiac positron emission tomography – computed tomography; PTFV1, P-wave terminal force in lead V1; SEC, spontaneous echo contrast.*

## Electrocardiography

### Introduction

An abnormal ECG may provide conduction disturbances and electrical remodeling. Studies analyzing ECG parameters defined LACM very heterogeneously. Associations with clinical outcomes (e.g., AF, ischemic stroke, major adverse cardiovascular events) and further imaging techniques evaluating LACM (e.g., abnormal echocardiographic parameters, low-voltage areas in electroanatomic mapping) were elaborated (Table 1).

### Fibrillatory Waves (F-Waves)

**FIGURE 2:**
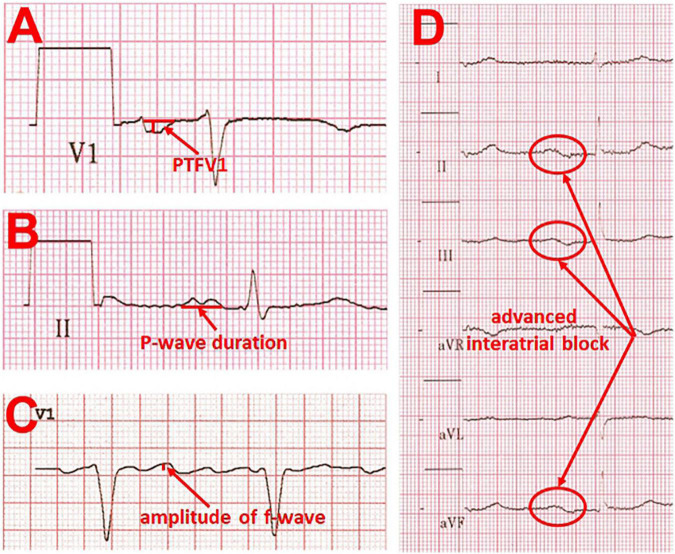
Examples of ECG changes that are indicative of left atrial cardiomyopathy. **(A)** pronounced P-wave terminal force in lead V1 ≤ –4,000 μV × ms (multiplying the amplitude of the second term of the P-wave by the width of this term). **(B)** Prolonged P-wave duration (≥120 ms) and double peaked morphology. **(C)** “Coarse” atrial fibrillation with an amplitude of > 0.1 mV. **(D)** Example of an advanced interatrial block, defined as P-wave duration ≥ 120 ms with simultaneous biphasic morphology in the inferior leads.

The analysis of fibrillatory waves (f-waves) may be suitable for detecting both electrophysiological and structural changes in the atria. “Coarse” AF defined as amplitude of f-wave in lead V1 ≥ 1 mm is associated with decreased LA appendage ejection fraction and decreased maximal emptying velocity (21). In addition, higher incidences of spontaneous echo contrast and thrombus in the LA appendage were observed in the presence of “coarse” AF (22). Furthermore, patients with paroxysmal, persistent, and permanent AF had different f-wave frequencies of 5.7 ± 0.7, 6.1 ± 0.8, and 6.2 ± 0.6 Hz, respectively (23). Besides, the frequency was lower in older than in younger patients. Moreover, the amplitude of the f-wave correlates strongly with the LA volume measured by echocardiography (21) (Figure 2C).

### P-Wave Indices

The P-wave represents the atrial depolarization of first the right atrium and then the LA, and is thus of particular interest with regard to atrial electrical remodeling (24). Interatrial excitation conduction disturbances via the Bachmann bundle can also be detected on the ECG. P-wave parameters comprise P-wave duration, P-wave dispersion, P-wave axis, P-wave voltage, P-wave area, interatrial block, and PTFV1 (24). First, several abnormal P-wave parameters are associated with the occurrence of AF. Second, they are predictive of ischemic stroke independent of AF, suggesting that they may reflect atrial remodeling irrespective of arrhythmogenesis (24). In addition, pathological P-wave parameters may be indicative of structural changes such as atrial enlargement (24).

### P-Wave Terminal Force in Lead V1

An important electrocardiographic marker for atrial remodeling is the PTFV1. The P-wave in lead V1 is usually biphasic, whereby the second, negative term of the P-wave represents the excitation propagation in the LA. The PTFV1 is determined by multiplying the amplitude of the second term of the P-wave by the width of this term. A PTFV1 ≤ −4,000 μV × ms is considered pathological (24, 25). In the past, several studies demonstrated an association between an abnormal PTFV1 and the occurrence of later AF (25–28). Moreover, an abnormal PTFV1 has also been associated with cryptogenic or cardioembolic stroke independent of the presence of AF (29–32). Kamel et al. (33) revealed that an abnormal PTFV1 was more strongly associated with the occurrence of stroke than with the occurrence of AF. In contrast, P-wave area and duration were not associated with stroke occurrence (30, 33). However, a meta-analysis demonstrated an association between abnormal PTFV1, P-wave duration, maximum P-wave area, and the risk of ischemic stroke (34).

In contrast, Yamamoto et al. (35) were not able to prove a difference in prevalence of an abnormal PTFV1 among patients with cardioembolic stroke, lacunar stroke and control subjects.

Abnormal PTFV1 is also associated with functional remodeling. First, it is associated with low LA appendage ejection velocity in transesophageal echocardiography (36). Second, LA strain measured by speckle tracking echocardiography as a marker of functional atrial remodeling is significantly reduced in the presence of an abnormal PTFV1 (37). There is a negative correlation between the PTFV1 and the LA conduction strain. Surprisingly, atrial fibrosis quantified by using Masson’s trichrome staining in atrial biopsies is significantly lower in patients with abnormal PTFV1. The results led to the suggestion that PTFV1 is possibly only a marker of electrical but not structural atrial remodeling (37) (Figure 2A).

### P-Wave Duration and Advanced Interatrial Block

However, the maximal P-wave duration was significantly prolonged in patients with cardioembolic and lacunar strokes. A P-wave duration ≥ 120 ms and an advanced interatrial block, defined as P-wave duration ≥ 120 ms with simultaneous biphasic morphology in the inferior leads (35), were more frequent in the ischemic stroke groups than in the control group. At the same time, the presence of a P-wave duration ≥ 120 ms and an advanced interatrial block was associated with a higher likelihood for subsequent stroke, particularly of cardioembolic origin. The occurrence of an advanced interatrial block by itself is also associated with both the incidence of AF and the incidence of thromboembolic events (25, 38). Higher body mass index (BMI) and older age correlate with prolonged P-wave duration and abnormal PTFV1, whereas higher blood pressure correlate with prolonged P-wave duration and right P-wave axis deviation (39) (Figures 2B,D).

### Novel Electrocardiography-Parameters

Recently, Müller-Edenborn et al. (40) conducted a study including patients with LA appendage thrombus to investigate an ECG-based diagnosis and staging of LACM using amplified P-wave (APW) analysis to stratify thromboembolic risk and cardiovascular outcome. Accordingly, LACM-stages were defined by APW duration, measured in digital ECG recordings from earliest beginning to latest activation in any of the 12 leads using standard amplification to 40–80 mm/mV and sweep speed of 100–200 mm/s, and P-wave morphology (40). No, moderate and extensive LACM were prevalent in 2.8, 21.1, and 76.1% of patients with LA appendage thrombi. The odds ratio for LA appendage thrombus was 24.6 (*p* < 0.001) per LACM-stage. Moreover, atrial contractile function, represented by LA appendage flow velocities, decreased with rising LACM-stages, while the occurrence of major adverse cardiovascular events increased (40). Abnormal APW duration is also associated with later onset of AF. An APW duration > 150 ms in patients with advanced heart failure with preserved ejection fraction (HFpEF) increased the risk for new-onset AF 10-fold (41). APW duration does not correlate with LA indexed volume but with recurrence-free survival after pulmonary vein isolation (PVI). Concordantly, an association has been demonstrated between electrophysiological evidence of LA low-voltage substrate and APW (41–43).

The duration of total atrial conduction time in non-invasive body surface electrocardiographic imaging correlated with the atrial activation time and extent of LA low-voltage substrate in endocardial contact mapping (44). Total atrial conduction time value of 148 ms identified an LACM with 91.3% sensitivity and 93.7% specificity, and the likelihood of arrhythmia recurrence after PVI was higher in patients with a total atrial conduction time > 148 ms (44).

Recently, a “novel artificial intelligence enabled ECG analysis” was performed to detect a possible LA myopathy in 613 patients with HFpEF (45). This method is based on a computer-assisted algorithm that analyzes data from raw 12-lead ECG signals and includes a statement about the probability of AF (45). Structural heart disease was more severe in patients with higher artificial intelligence probability of AF, with increased left ventricular hypertrophy, larger LA volumes, and decreased LA reservoir and booster strain in echocardiography. Each 10% increase in artificial intelligence probability resulted in a 31% higher risk of developing new-onset AF among patients with sinus rhythm and no prior AF. In the total population, each 10% increase in artificial intelligence probability led to a 12% higher risk of death (45).

Holter ECG may also provide evidence of LACM since excessive atrial premature complexes in patients with an ischemic stroke or a transient ischemic attack correlate with LA remodeling (46).

## Echocardiography

### Transthoracic Echocardiography

#### Introduction

On the one hand, the positive correlation between LA enlargement and the occurrence of adverse cardiovascular outcomes is well known (9, 47). On the other hand, an enlarged LA is also associated with the incidence of AF (9). Echocardiography is the imaging technique of choice for the screening and follow-up of patients with abnormal LA morphology and function because of its widespread use, non-invasiveness and cost efficiency (9). Thus, echocardiography may be useful in detection of LACM. In studies investigating the utility of echocardiography, LACM was defined by demonstrating an association of abnormal LA size with mainly clinical outcomes such as AF, AF burden, recurrence of AF after ablation and ischemic stroke (Table 1).

#### Left Atrial Size

**FIGURE 3:**
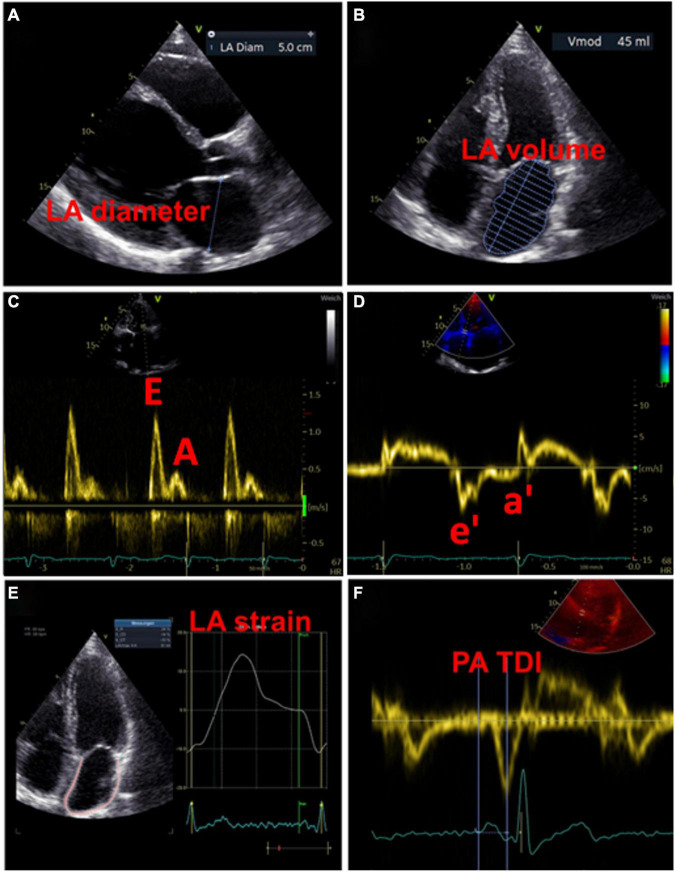
Examples of echocardiographic measurements for the detection of left atrial cardiomyopathy. **(A)** Measurement of diameter in left atrial diastole. **(B)** Measurement of left atrial volume in left atrial diastole. **(C)** Transmitral inflow profile in left ventricular diastole: the first wave represents the E wave (passive inflow of blood into the left ventricle), the second wave represents the A wave (active contraction of the left atrium). **(D)** Tissue Doppler imaging of the movements of the left ventricular myocardium, in combination with the measurements from **(C)** the function of the atrium and the left ventricular end-diastolic pressure can be estimated. **(E)** Strain analysis of the left atrium. **(F)** Measurement of the PA-TDI interval from the beginning of the P-wave (as the onset of electrical activity of the atrium) to the peak of the a′-wave (mechanical response of atrial contraction).

A widely used parameter for the estimation of LA size is the LA diameter (Figure 3A). In the AFFIRM study, an increasing LA diameter correlated with recurrent AF, but not with the risk of stroke (48). However, a meta-analysis revealed an association between a large LA diameter and the incidence of stroke and thromboembolic events (49). Moreover, large LA diameter and LA volume index were both demonstrated as predictive parameters for major adverse cardiovascular and thromboembolic events, particularly in young patients without AF (49, 50). The LA volume index is more precise and therefore more suitable for estimating atrial size (Figure 3B). Increased LA volume index has been described as a potential early marker of myocardial dysfunction and is often present in patients with AF, with increasing frequency in a higher AF burden (51, 52). Besides, the minimal LA volume strongly correlates with the incidence of new-onset AF and major adverse cardiovascular events compared to the maximal LA volume that seems to have no predictive effects (53–56).

In addition to abnormalities in LA size that represent structural remodeling, assessment of atrial function may provide further important indicators for the presence of LACM. Both enlarged LA and decreased LA emptying fractions are common phenomena in patients with AF, with a negative correlation between LA size and emptying fraction (51). Recently, Eichenlaub et al. evaluated the LA emptying fraction in patients with AF for the diagnosis of LACM and prediction of arrhythmia recurrence after PVI (57). An LACM was defined as a LA low-voltage area ≥ 2 cm^2^ at 0.5 mV threshold on endocardial contact mapping. Patients with LACM had lower LA emptying fractions than patients without LACM (27 vs. 41%, *p* < 0.0001) (43). Furthermore, LA emptying fraction < 34% was a significant predictor of both LACM, with an area under the curve of 0.846, and recurrence of arrhythmia after PVI (57).

#### Doppler Echocardiography

Additionally, the assessment of the LA function is possible by means of pulsed wave doppler measurements and tissue doppler imaging. Impaired LA function may be indicative of LACM, which was equated with clinical outcomes such as AF and AF burden in the studies that used doppler echocardiography (Table 1).

In the past, numerous echocardiographic parameters relating transmitral blood flow and diastolic myocardial motion have been investigated for association with LA function and clinical events (51, 52, 58, 59). However, no parameter and corresponding cut-off value have yet been identified as suitable for the diagnosis of LACM.

Whereas LA conduction function (represented by transmitral E-wave velocities) increases with higher AF burden, there is an opposite effect in LA contractile function (represented by transmitral A-wave velocities and mitral annular tissue Doppler a’ velocities).

(51). There is an association between an increased ratio of the early [E] and late [A] diastolic filling waves and the risk of AF. Moreover, a U-shaped relationship between peak A-wave velocity and AF risk was described (59).

A reduced mitral annular “e”-wave velocity and an increased E/e ratio provide signs of impaired LV relaxation. There is evidence that the latter parameter is also suitable to assess LA function and pressure (52) (Figures 3C,D).

#### Total Atrial Conduction Time Assessed by Tissue Doppler Imaging Duration

Total atrial conduction time assessed by tissue doppler imaging duration, representing the echocardiographic derived total atrial conduction time, is an auspicious marker of both structural and electrical atrial remodeling, which is measured during sinus rhythm as the time interval between the onset of the P-wave in lead II on surface ECG and the peak of the A′ wave on tissue Doppler imaging (TDI) tracing of the lateral LA wall on echocardiography (60) (Figure 3F). Prolonged PA-TDI duration correlates with new-onset AF, post-operative AF and AF recurrence after rhythm control interventions (60). In patients with AF, assessment of thromboembolic risk has been improved by adding the PA-TDI duration value. To date, standard reference values for PA-TDI duration have not been established. Nevertheless, if each echocardiography laboratory determines its own normal values by routinely obtaining the PA-TDI value, risk assessment for AF-related outcomes may be improved (60).

#### 3-Dimensional and 4-Dimensional Echocardiography

In the last few years, 3-dimensional and 4-dimensional echocardiography have improved the options of LA volume measurements. Studies using 3-dimensional and 4-dimensional echocardiography defined LACM by abnormal LA wall delayed-enhancement (Table 1). Recently, in a subanalysis of the LOOP trial investigating LA fibrosis by 4-dimensional echocardiography, an association of minimal LA volume, LA emptying fractions, and LA reservoir strain with LA late gadolinium enhancement measured by cardiac magnetic resonance imaging (MRI) was observed (56). LA emptying fractions had the strongest effect on predicting high LA late gadolinium enhancement and therefore LA fibrosis (56).

#### Speckle-Tracking Echocardiography

In recent years, speckle-tracking echocardiography has become a popular method for detecting early myocardial deformation by assessing the tissue movement (9). LA strain and strain rate imaging provide insights into functional remodeling of the atrium (Figure 3E). Studies evaluating the utility of speckle-tracking echocardiography defined LACM mainly by clinical definitions (e.g., AF, AF burden, AF recurrence, thromboembolic events) or by comparison with abnormal findings in electroanatomic mapping (Table 1).

In patients with severe mitral regurgitation, there was a strong correlation between the impairment of LA longitudinal deformation, as evaluated by the global peak atrial longitudinal strain, and the extent of LA fibrosis and remodeling (61). A reduced global longitudinal LA strain was described in patients with AF and presents a predictor for thromboembolism (62–64). There is evidence that a higher AF burden (≥10%) is particularly associated with decrease in global longitudinal LA strain, which correlates with mean LA strain measured by mapping and may be improved after AF ablation (65). In the study of Eichenlaub et al. (57), a LA longitudinal strain rate < 23.5% predicted LACM, defined as LA low-voltage area ≥ 2 cm^2^ at 0.5 mV threshold on endocardial mapping, with an area under the curve of 0.878, a sensitivity of 92.3% and specificity of 82.4%. In patients with LACM, LA strain rate during reservoir phase was significantly lower (15.2 vs. 29.4%, *p* < 0.0001) and showed a linear correlation with LACM amount (43).

Moreover, the addition of global longitudinal LA strain and LA volume index to the CHA2DS2-VASc score improves also the prediction of hospitalization and/or mortality (62).

Even in patients with sinus rhythm, restricted LA diastolic function, represented by the peak atrial longitudinal strain and the LA stiffness index, was strongly associated with low amplitude voltage areas measured by endocardial mapping (66).

By means of speckle-tracking echocardiography it is possible to specify the functional remodeling in different regions of the atrium. For example, a declined LA lateral wall longitudinal strain was found to be a predictor of arrhythmia recurrence after AF ablation (67). In patients with amyloidosis, the septal LA strain and strain rate were overall lower (68). Furthermore, lateral and septal LA strain rates were decreased in patients with heart failure compared to those without (68).

A higher LA mechanical dispersion, defined as the standard deviation (SD) of time to peak positive strain corrected by the R-R interval (SD-TPS, %), was described in patients with AF than in those without (64). Besides, SD-TPS was associated with the incidence of new-onset AF (64). The LA mechanical dispersion was also measured in patients with paroxysmal AF undergoing PVI. The SD-TPS was significantly higher in patients with low voltage areas, measured by mapping, and was simultaneously an independent predictor for low voltage areas (69).

### Transesophageal Echocardiography

**FIGURE 4:**
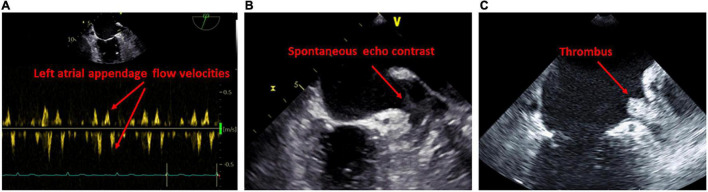
Findings on transesophageal examination suggestive of left atrial cardiomyopathy. **(A)** Reduced blood flow in the ostium of the left atrial appendage. **(B)** Evidence of spontaneous echo contrast in the left atrial appendage. **(C)** Evidence of thrombus in the left atrial appendage.

An important advantage of transesophageal echocardiography is the more precise assessment of the LA appendage. There is limited evidence on the correlation of transesophageal abnormalities and LACM which has been defined mainly by fibrosis determination using delayed-enhancement MRI. It was demonstrated that patients with LA appendage thrombus had a higher amount of LA fibrosis, diagnosed by late gadolinium enhancement MRI, in comparison to patients without thrombus (70). LA fibrosis was even higher in patients with spontaneous echo contrast than in those without. In addition, patients with high atrial fibrosis were more likely to have both thrombus and spontaneous echo contrast in the LA appendage (70). It is common knowledge that reduced LA/LA appendage flow dynamics and increased LA size are risk factors for the occurrence of thrombus and spontaneous echo contrast (71) (Figure 4).

## Cardiac Magnetic Resonance Imaging

### Introduction

Using MRI, LA volume and LA strain can be detected. Decreased LA strain measured by MRI is present in patients with AF (72). A modest correlation between speckle-tracking echocardiography and MRI obtained LA volume and LA strain has been observed (73, 74). There are systematic differences in measurements that should be taken into account as MRI measurements reveal higher values (73, 74).

### Delayed-Enhancement Magnetic Resonance Imaging

Over recent years, delayed-enhancement MRI became the key method to detect atrial fibrosis. Studies aiming to define LACM by pathological delayed-enhancement MRI focused particularly on clinical definitions (e.g., AF, ischemic stroke, major adverse cardiovascular events) as well as on further imaging definitions (e.g., abnormal echocardiographic parameters, low-voltage areas in electroanatomic mapping) (Table 1).

**FIGURE 5:**
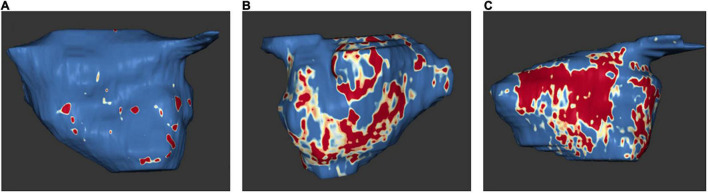
Examples of MRI examinations of the left atrium: left atrial tissue fibrosis based on 3D delayed enhancement magnetic resonance imaging scans. Normal left atrial wall is displayed in blue, fibrotic changes are in red and white. Amounts of fibrosis as a percentage of the total left atrial wall volume. **(A)** Utah stage 1 (1%). **(B)** Utah stage 3 (27%). **(C)** Utah stage 4 (36%). *With the friendly support of Dr. Misagh Piran (Herz- und Diabeteszentrum Nordrhein-Westfalen, Ruhr-Universität Bochum)*.

In 2009, Oakes et al. established the Utah stage model to quantify LA fibrosis (75). Based on this model, four levels of severity are classified: Utah I, defined as ≤ 5% LA wall enhancement, Utah II, 5–20%, Utah III, 20–35%, and Utah IV, >35% (12) (Figure 5).

High correlations were discovered between delayed-enhancement MRI and histology from surgical biopsies for LA structural remodeling including interstitial and fatty fibrosis as well as total fibrosis and fat (76, 77). Native T1 corresponded with the extent of fibrosis from MRI and histology (76). Moreover, LA wall enhancement appeared to be greater in patients with AF than in patients without AF (77).

Regions of scar in the LA identified by delayed-enhancement MRI were noted to be associated with low-voltage areas in endocardial mapping (78).

A higher amount of LA late gadolinium enhancement is associated with decreased LA function as well as decreased LV diastolic function since significant correlations were described between LA late gadolinium enhancement and both LA ejection fraction and echocardiographic LV septal e′ and septal E/e′ (79). A correlation between delayed-enhancement and reduced LA function has also been demonstrated by speckle-tracking echocardiography. There was an inverse effect between the extent of LA wall fibrosis measured by delayed-enhancement MRI and LA strain and strain rate in speckle-tracking echocardiography, particularly LA midlateral strain and strain rate (80). Interestingly, patients with persistent AF presented with more fibrosis and less midseptal and midlateral strain compared to patients with paroxysmal AF (80). In general, the risk for new-onset atrial arrhythmias is higher by increasing amount of LA late gadolinium enhancement (79).

Patients with previous strokes and high-risk patients for stroke, reflected by a high CHA2DS2-VASc score, had a significantly higher proportion of LA fibrosis in delayed-enhancement MRI. LA fibrosis was an independent predictor of cerebrovascular events and significantly increased the predictive power of the CHA2DS2-VASc score (81). Rising Utah stage and more intense LA late gadolinium enhancement were associated with increased risk of major cardiovascular events, predominantly due to the increased risk of stroke or TIA (82). Interestingly, patients with ESUS present with similar amount of atrial fibrosis compared to patients with AF which supports the hypothesis that fibrosis is a major risk factor for ischemic stroke and LACM (83).

The extent of structural LA remodeling measured by delayed-enhancement MRI seems to be independent of the AF type (lone AF or non-lone AF) (84). The outcome after AF ablation was demonstrated to be significantly dependent on the degree of structural LA remodeling, with worse outcome at increasing Utah stage (84). With an increasing level of delayed-enhancement, AF recurrence after ablation occurred more frequently (75). Therefore, pre-ablation LA fibrosis assessment by delayed-enhancement MRI may predict the outcome (75). Another study examining the impact of LA fibrosis on the outcome after AF ablation demonstrated that the risk of recurrent arrhythmias appeared at higher LA fibrosis grades detected by delayed-enhancement MRI (77). The presence of higher LA fibrosis grades was also the best predictor for successful ablation, while increased LA volume and persistent AF had no predictive effect (77).

The DECAAF study evaluated the impact of LA fibrosis measured by delayed-enhancement MRI on the outcome after AF ablation (85). Among 329 patients undergoing AF ablation, the extent of LA fibrosis estimated by delayed-enhancement MRI correlated significantly with the recurrence of arrhythmia (85). The prospective, randomized, multicenter DECAAF II study provides further insights into the value of preprocedural LA fibrosis estimation by delayed-enhancement MRI in patients with persistent AF experiencing AF ablation (86). The results, which were presented at the ESC Congress 2021 but not yet published, demonstrated that fibrosis-guided ablation was not superior to conventional PVI in reducing recurrence of AF (86).

### 4-Dimensional Flow Magnetic Resonance Imaging

4-dimensional flow MRI yields 3-dimensional volume sets over time (4-dimensional). This enables a precise quantitative evaluation of cardiovascular blood flows (87). In studies analyzing LA blood flow velocities, LACM has been equated with clinical definitions (e.g., AF burden), and further imaging definitions (e.g., abnormal echocardiographic parameters) (Table 1).

Contrasting data exist on blood flow velocities in patients with AF. On the one hand, there is evidence that patients with AF in history have a similar blood flow in the normal range at sinus rhythm compared to age-matched controls in measurements with 4-dimensional flow MRI (88, 89). On the other hand, there are findings that AF patients present with lower LA mean velocities and more often LA stasis compared to controls (90, 91).

Nevertheless, patients with persistent AF have a significant lower LA blood flow compared to AF patients at sinus rhythm (88). Moreover, a negative correlation was seen between increased CHA2DS2-VASc score and decreased mean LA velocity (89, 91). Interestingly, a correlation was described between LA blood flow indices, age and LA volume, but not with left ventricular ejection fraction (91).

## Cardiac Computed Tomography

### Cardiac Computed Tomography

It is well known that cardiac CT can be used for precise measurement of atrial volumes (9). A high correlation between LA volume index obtained by CT and speckle-tracking echocardiography has been observed (92). Moreover, LA volume index measured by CT is a predictor of AF recurrence after ablation (93).

Furthermore, it has recently been demonstrated that CT can also be applied for strain measurements. LA strain measured by CT correlates strongly with strain measurements derived from speckle-tracking echocardiography (94–96).

In addition, structural changes of the atria can be detected by CT. Image attenuation ratio collected by CT predicts LA low-voltage areas in electroanatomic mapping which suggests that CT can help to assess fibrosis when contraindications for MRI exist (97).

### Cardiac Positron Emission Tomography – Computed Tomography

In recent years, an association between atrial 18F-fluorodeoxyglucose activity in positron emission tomography – CT (PET-CT), previous ischemic stroke and LACM in non-AF individuals was demonstrated (98). LACM was defined either by increased atrial 18F-fluorodeoxyglucose uptake itself or by clinical outcomes, such as AF, AF burden, and stroke (Table 1).

Enhanced atrial activity was associated with ischemic stroke and LACM (98). In addition, higher atrial uptake of 18F-fluorodeoxyglucose was observed in patients with AF, whereas persistent AF showing higher atrial uptake than paroxysmal AF (99). Persistent AF is particularly associated with right atrial maximum standard uptake value and LA volume. Moreover, the right atrial target-to-background ratio of maximum standard uptake value to blood pool activity seems to be greater in patients with persistent AF than in those with paroxysmal AF (99). Data about the value of 18F-fluorodeoxyglucose PET-CT in patients with suspected LACM are very rare. 18F-fluorodeoxyglucose PET-CT could be useful to explore local atrial inflammation and to stratify the risk of subsequent stroke by monitoring disease activity in patients with AF. Nevertheless, a widespread use to detect LACM cannot be implemented. In oncologic patients undergoing PET-CT, this diagnostic tool can be used supplementally when additional LACM is suspected.

## Electroanatomic Mapping and Ablation

### Introduction

Electroanatomic mapping is another valuable method providing additional insights into patients with presumed LACM. There a numerous studies aiming to define LACM by the presence of low-voltage areas and their association with clinical definitions (e.g., AF, AF recurrence, ischemic stroke) and with further imaging definitions (e.g., abnormal echocardiographic parameters) (Table 1).

### Electrophysiological Findings in Left Atrial Cardiomyopathy

Patients with paroxysmal lone AF exhibit bi-atrial abnormalities including structural remodeling, conduction disorders, and sinus node dysfunction (100). Electrophysiological findings in these patients were enlarged atrial volumes, increased effective refractory period at multiple sites, increased conduction time along linear catheters, increased bi-atrial activation time, decreased conduction velocity, more frequent fractionated electrograms, increased corrected sinus node recovery time, and lower voltage (100).

### Low-Voltage Areas

**FIGURE 6:**
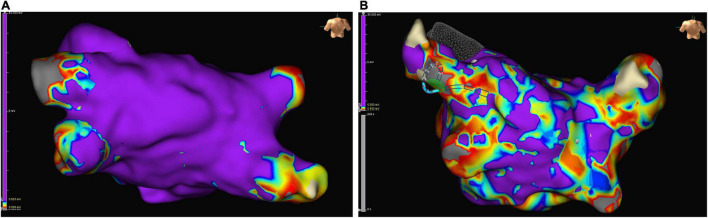
Examples of low-voltage areas in endocardial mapping of left atrium in patients with sinus rhythm who underwent pulmonary vein isolation: normally conducting atrial myocardium is colored purple, low voltage areas (defined as zones with an amplitude of electrical perception of ≤ 0.5 mV) are colored differently. **(A)** Example of a left atrium almost without low voltage areas. **(B)** Example of a severely diseased left atrium with low voltage areas > 10%.

Patients with non-focal LA tachycardia presented with a high proportion of low-voltage areas in endocardial mapping, providing evidence for possible LACM (101) (Figure 6). An analysis of patients with fibrotic LACM revealed that severe fibrotic areas increased and maximum LA voltage decreased, with growing severity of fibrotic LACM (101).

An inverse relationship was found between lower mean LA voltage and higher CHA2DS2-VASc score (102). A significant association has been described between electroanatomic LA remodeling and the risk of stroke in patients with AF (102). In AF patients with previous stroke, low-voltages area as well as pre-existing silent cerebral ischemia were significantly larger detected by cerebral MRI after PVI, even after adjustment of CHA2DS2-VASc score (103). Whereas the mean LA volume/body surface area, particularly anterior LA, was greater, the LA endocardial voltage was lower in patients who suffered stroke (102).

#### Localization of the Low-Voltage Areas

Of particular interest is also the localization of the low-voltage areas. A relationship was described between anterior low-voltage areas and macro-re-entry mechanism by forming a conducting channel between the lower pole of the LA scar and the mitral valve annulus (101). Although the distribution of LA fibrosis is often variable, it is usually more pronounced anteriorly (104). Anterior severely fibrotic areas have been found to be more frequent and larger than posterior severely fibrotic areas (104). The knowledge that mainly the anterior LA is affected allows an individualized ablation approach that could complement the usual strategies (101).

#### Quantification of Low-Voltage Areas and Clinical Impact

A high percentage of low-voltage areas as well as fibrosis areas in the LA predicts recurrence of AF after catheter ablation (104–108). In patients with multiple ablations, AF recurrence rate was significantly higher in patients with low-voltage areas than without (36 vs. 6%, *p* < 0.001) (106). The size of fibrotic areas, fibrosis in the LA as well as in the right atrium, and decreased maximum voltage has negative impact on the ablation outcome (104). Furthermore, LA fibrosis areas correlated significantly with larger LA size, decreased ejection fraction, and higher C-reactive protein levels (107).

The modified APPLE score (one point for age ≥ 65 years, persistent AF, impaired estimated glomerular filtration rate ≤ 60 mL/min/1.73 m^2^, LA volume ≥ 39 mL/m^2^, and LA ejection fraction < 31%) may be possible to estimate the success rate of catheter ablation (109).

Knowing that the degree of low-voltage areas is a key determinant of ablation success, individualized ablation approaches may be beneficial. Ablation of lesions with prominent activation features within/at the margins of low-voltage areas in addition to PVI might be more effective than the conventional strategy of PVI solely in patients with persistent AF (110). In contrast, PVI alone appears to be sufficient for the treatment of patients with LA low-voltage < 10%, as no significant difference in success rate was demonstrated between patients with low-voltage who underwent PVI alone and patients who required PVI + selective low-voltage ablation (110).

Most studies evaluating the benefit of electroanatomic mapping for the assessment of LACM include patients with AF undergoing catheter ablation. There is strong evidence that patients with AF have both more low-voltage areas and severe fibrotic areas reflecting electrical and structural remodeling of the atrium (100–110) (Figure 6).

## Biomarkers

### Introduction

A number of circulating biomarkers have been proposed to estimate atrial remodeling and LACM (9, 111). Studies analyzing biomarkers defined LACM by clinical definitions (e.g., AF, AF burden, AF recurrence, stroke) as well as by further imaging definitions (e.g., low-voltage areas in electroanatomic mapping, abnormal echocardiographic parameters) (Table 1).

### Marker of Inflammation

Atrial inflammation is discussed as a key factor for atrial fibrosis and the risk of AF (112). The metabolic syndrome is a well-known risk factor for inflammatory processes. Obesity, hypertension, and diabetes mellitus predispose to AF (9). Epicardial adipose tissue secretes proinflammatory and profibrotic cytokines as well as profibrotic microRNA which may promote the development of both AF and inflammation-induced LACM (112, 113). Proinflammatory IL-6 levels, MMP-9/TIMP-1 ratio as well as NF-AT3 and NF-AT4 mRNA and protein expression were significantly increased in patients with AF, particularly in persistent AF (114, 115). High sensitivity C-reactive protein was described as a predictor of arrhythmia recurrence after AF ablation (116).

Increased TGF-ß1 levels in monocytes were seen in patients who exhibited extensive low-voltage areas on endocardial mapping (117). Moreover, it has been demonstrated that higher serum TGF-ß1 levels correlated with the presence of AF and arrhythmia recurrence after AF ablation (118, 119). With increasing AF duration, serum TGF-ß1 levels declined (114). However, the predictive value of serum TGFß-1 level for the recurrence of arrhythmias seems to be only in patients with non-paroxysmal AF (120), and not all studies could demonstrate a risk for AF (121). The same applies to the concordance of serum TGF-ß1 levels and echocardiographic parameters. On the one hand, there is evidence about an inverse relationship between serum TGF-ß1 level and LA diameter (114), but on the other hand there are also findings that could not prove an association (119).

### Marker of Fibrosis

Furthermore, it is suggested that specific fibrosis markers may be related to atrial remodeling. Surprisingly, Type III procollagen N terminal peptide, galectin-3, fibroblast growth factor 23, and type I collagen C terminal telopeptide were not predictors of arrhythmia recurrence after AF ablation (105). Type III procollagen N terminal peptide also revealed merely a slight positive trend with regard to AF risk (121). However, the combination of circulating biomarkers reflecting excessive myocardial collagen type-I cross-linking and deposition shows a predictive effect on higher AF prevalence, incidence, and recurrence after ablation (122). In addition, a negative correlation was described between galectin-3 and echocardiographic parameters assessing the LA, including LA volume and LA strain rate (123). ST-2 and apoptotic microparticles are also associated with increased LA volume index (124). Besides, galectin-3 and soluble ST-2 were significantly higher in patients with stroke and AF compared to patients with stroke without AF (125).

### Atrial Peptides

N-terminal pro-B-type natriuretic peptide is a well-known indicator of congestive heart failure due to volume overload and myocardial damage. N-terminal pro-B-type natriuretic peptide showed a strong correlation with echocardiographic parameters of LA remodeling and dysfunction, and was a significant but weak predictor of AF (125). Moreover, there is an association with AF burden (114). An inverse relationship was reported between higher levels of endothelin-1, N-terminal pro-B-type natriuretic peptide, troponin I and lower LA reservoir and contractile strain, suggesting that LA myopathy is associated with persistent congestion (126, 127). Oldgren et al. evaluated the ABC-stroke score, derived from the ARISTOTLE study, including age, biomarkers (N-terminal pro-B-type natriuretic peptide and high-sensitivity cardiac troponin), and clinical history (prior stroke) (128, 129). In anticoagulated patients with AF, the biomarker-based ABC stroke score was well applicable and generally more suitable than the CHA2DS2-VASc and ATRIA stroke scores (128).

N-terminal pro-A-type natriuretic peptide is a hormone released by the atria in response to increased atrial tension. Recently, Seewöster et al. introduced the novel biomarker-based ANP score (one point for age ≥ 65 years, N-terminal pro-A-type natriuretic peptide > 17 ng/mL, and persistent AF) which significantly predicted low-voltage areas in patients undergoing AF ablation (130).

### Aldosterone and Renin

In patients with persistent AF, aldosterone levels and aldosterone/renin index decreased significantly after successful ablation of AF. It is worth noting that aldosterone and renin levels did not interact with duration of AF, LA diameter, mean heart rate, systolic blood pressure, age, New York Heart Association class, or left ventricular ejection fraction (131).

### Marker of Immunothrombosis

The close interactions between the immune system and the coagulation cascade can be summarized under the term “immunothrombosis.” On the one hand, the immune system initiates coagulation; on the other hand, local coagulation has pro-inflammatory and pro-fibrotic effects (132, 133). Thus, immunothrombosis might be an underlying mechanism of atrial remodeling and AF (134). In the last decades, several coagulation markers were described in patients with AF, with both impact on thromboembolism and bleeding risk. For example, abnormal levels of von Willebrand factor, D-dimer, growth differentiation factor 15, soluble p-selectin, coagulation factor Xa and endothelial nitric oxide synthase indicate endocardial remodeling (135–140). Nevertheless, a recent study was not able to demonstrate a correlation between immunothrombosis markers and incident AF after adjustment for cardiovascular risk factors (134). This may indicate that inflammation and immunothrombosis may be associated with AF by other cardiovascular risk factors, rather than AF *per se* (134).

### Conclusion

Circulating biomarkers can provide evidence of inflammatory and fibrosis pathways in the atrium. However, there are sometimes conflicting results, and the inflammation- and fibrosis-related biomarkers are not specific. Therefore, the clinical value in the assessment of LACM is currently unclear, and routine screening seems questionable. However, future research investigating these pathways has potential, and treatment options could be identified that specifically intervene in the inflammatory and fibrosis regulatory circuits.

In conclusion, Table 2 summarizes laboratory parameters that have been used as markers of LACM in different studies.

**TABLE 2:** Overview of selected biomarkers with association to LACM, AF, and thromboembolic events.

Laboratory parameter	Up- or downregulation is associated with AF incidence	Associations	References
Aldosterone	↑	AF, AF recurrence after cardioversion	(131)
Aldosterone/renin	↑	AF, AF recurrence after cardioversion	(131)
Coagulation factor Xa	↑	AF, atrial remodeling	(139)
CRP	↑	AF recurrence after ablation	(116)
D-dimer	↑	Thromboembolic events in patients with AF	(140)
Endothelial nitric oxide synthase	↓	AF	(138)
Endothelin-1	↑	Abnormal echocardiographic parameters	(126, 127)
Galectin-3	↑	AF, stroke, abnormal echocardiographic parameters	(123, 125)
Growth differentiation factor 15	↑	AF	(137)
IL-6	↑	AF, AF burden	(114)
MMP-9	↑	AF, AF burden	(114)
MMP-9/TIMP-1	↑	AF, AF burden	(114)
NF-AT3	↑	AF, AF burden	(115)
NF-AT4	↑	AF, AF burden	(115)
N-terminal pro-A-type natriuretic peptide	↑	Low-voltage areas in electroanatomic mapping	(130)
N-terminal pro-B-type natriuretic peptide	↑	AF, AF burden, abnormal echocardiographic parameters	(114, 125–129)
Type III procollagen N terminal peptide	↑	AF	(121)
Soluble p-selectin	↑	AF	(135, 136)
Soluble ST-2	↑	AF, stroke, abnormal echocardiographic parameters	(124, 125)
TGF-ß1	↑	AF, AF burden, AF recurrence after ablation, low-voltage areas in electroanatomic mapping, abnormal echocardiographic parameters	(114, 117–121)
Troponin I	↑	Abnormal echocardiographic parameters	(126, 127)
Von Willebrand factor	↑	AF, stroke	(135, 136)

*AF, atrial fibrillation; LACM, atrial cardiomyopathy; CRP, C-reactive protein; IL-6, interleukin 6; MMP-9, matrix metalloproteinase 9; NF-AT, nuclear factor of activated T cells; ST-2, suppression of tumorigenicity 2; TGF-ß1, transforming growth factor β 1; TIMP-1, tissue inhibitor of metalloproteinase 1.*

## Risk Factors

**FIGURE 7:**
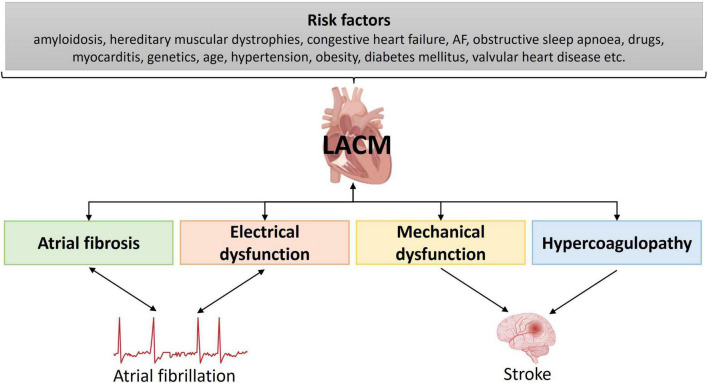
Illustration of the underlying risk factors for left atrial cardiomyopathy. AF, atrial fibrillation; LACM, left atrial cardiomyopathy.

Several risk factors for the development of AF as well as structural and electrical remodeling in terms of a possible LACM have been described. LACM may occur as a result of amyloidosis, hereditary muscular dystrophies, congestive heart failure, AF, obstructive sleep apnea, drugs, alcohol, myocarditis, genetic repolarization disturbances, aging, hypertension, obesity, diabetes mellitus, valvular heart disease (9, 127, 141–143) (Figure 7).

The newly developed AF-SCORE (+1 point for age ≥ 60 years and additional points for female sex [+1] and AF-persistency [+2]) enabled a good discrimination to identify fibrotic LACM and predicted arrhythmia-freedom after PVI (144). A low AF-SCORE ≤ 2 was more frequently observed in patients with paroxysmal AF of any age and in younger patients with persistent AF, irrespective of sex, and is associated with better PVI-only outcomes (144).

## Critical Considerations on the Concept and Diagnosis of Left Atrial Cardiomyopathy

### Diagnosis

The assessment of electrical, mechanical, and structural LA dysfunction seems feasible due to several diagnostic tools. However, single diagnostic procedures might not be sufficient for the diagnosis of LACM. In particular, because the diagnosis of LACM is challenging, a combination of parameters associated with LACM should be sought.

### Clinical Endpoints

Most data were generated from studies that included patients with AF and/or with stroke. Because it is not yet completely understood how LACM can be diagnosed, there are very few studies that directly address LACM. Therefore, the parameters found to define LACM are vague and may not be specific. Focusing on patients with AF could be critical because it disregards subclinical LACM before the onset of AF.

### Heterogeneity of Studies

It should also be considered that the comparability of the studies may not be given, because LACM and other parameters (e.g., low-voltage areas) were not defined standardized. Another critical consideration is that most studies equate LA myopathy with atrial cardiomyopathy in general. Perhaps, however, an atrial cardiomyopathy of the right atrium is different from an atrial cardiomyopathy of the LA. Unqualified transferability from the LA to the right atrium is not reasonable. Further investigation is required. Many studies concerning LACM analyzed only atrial fibrosis. However, fibrosis can also result from degenerative processes, increasing age, and other comorbidities. It is likely that LACM consists of a compound entity.

### Therapeutic Consequences

Differentiation in terms of severity of LACM is important to identify high-risk patients. Possible differentiation could be achieved by the presence or absence of risk factors. It is uncontroversial that the presence of an LACM with concomitant multiple cardiovascular risk factors poses a high risk for major adverse cardiac events. However, it becomes more complicated in the absence of risk factors. This raises the question of whether a patient without risk factors but with evidence of LACM would benefit from therapy. Further outstanding issues address therapy and prevention strategies, which might include more aggressive rhythm control, close follow-up, or the prescription of oral anticoagulation. Conversely, a patient without risk factors also has a lower cardiovascular risk. Thus, it is interesting and indeed clinically very relevant to consider the impact of LACM by itself on stroke risk.

### Left Atrial Cardiomyopathy and Atrial Fibrillation

The most extreme assumption would be that it is not AF itself that causes stroke but AF-associated LACM (1). Stroke prevention might become the key to therapeutic options. On the one hand, the identification of high-risk patients without previous AF is important, on the other hand, it could be evaluated whether patients with AF need oral anticoagulation at all. These considerations could also have implications for the use of oral anticoagulants after AF ablation. Conversely, there is also the possibility that patients with oral anticoagulants are not adequately managed (1).

Moreover, the presence of AF or the occurrence of thromboembolic events could already represent a later disease course of LACM. It is often assumed that LACM precedes the incidence of AF. Hence, diagnostic criteria of LACM are subsequently derived from predictors for the occurrence of AF. However, pre-existing AF may also trigger atrial remodeling. The landmark concept “AF begets AF” probably does not adequately address the diagnosis of LACM since there might be a bidirectional relationship between AF and atrial fibrosis (9). It is unknown why there are such large interindividual differences. Why does one patient persist in paroxysmal AF for several years while another progresses to persistent AF within a few weeks? Surprisingly, even patients with an extremely high amount of atrial fibrosis can present with paroxysmal instead of persistent AF (10). This “chicken and egg” situation, whether AF is a symptom of LACM or a trigger for LACM, cannot be conclusively resolved. However, it is important to be aware that one-sided considerations and assumptions can lead to a distortion of the complex clinical pattern of LACM.

### How to Diagnose Left Atrial Cardiomyopathy

Another reason for this elusive clinical pattern is the current classification of LACM, which is based on atrial histopathology (9). Although the classification has enormously helped to define LACM histopathological, it requires biopsies, which is difficult to implement and not reasonable in the clinical setting. There is hope that with improved clinical diagnostics, an improved classification might be possible (1). To date, there is no recommendation for the clinical diagnosis of LACM. In addition, the current classification does not yet provide any guidance for therapies and prevention strategies (9). Again, the question arises whether the management of patients can be improved by clinical diagnostics and clinical classification. This present review attempts to take a first step in this direction.

Randomized controlled trials could help to improve diagnostic and therapeutic options in the presence of LACM. This could also result in helpful advises for patient follow-up.

## Proposal for a Diagnostic Algorithm: How to Diagnose Atrial Cardiomyopathy?

From the studies discussed in this review, a complex picture emerges of a disease that is difficult to diagnose. The main methods used to diagnose LACM are cardiac MRI, electrophysiological investigations, echocardiography, and ECG. In contrast, there are few studies with cardiac CT and PET-CT and many but inconsistent studies with biomarkers.

**FIGURE 8:**
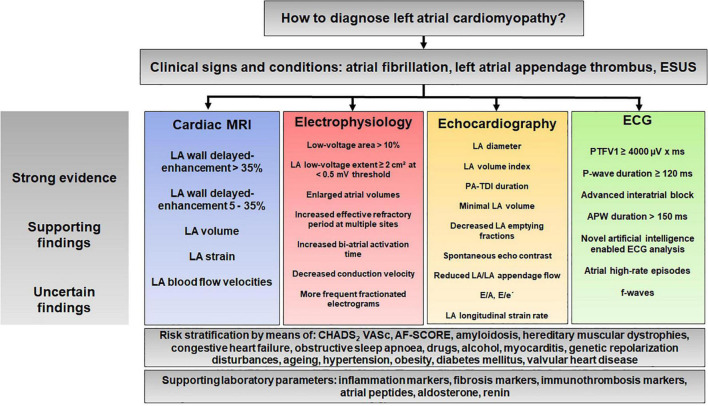
Proposal for a diagnostic algorithm for atrial cardiomyopathy based on the results of previous studies. APW, amplified P-wave; ESUS, embolic stroke of undetermined source; LA, left atrium/left atrial; PA-TDI, total atrial conduction time assessed by tissue doppler imaging; PTFV1; P-wave terminal force in lead V1.

Although many questions remain, we would like to propose here a diagnostic algorithm that summarizes this review (Figure 8). This algorithm is intended to illustrate the complexity of the diagnostic process and may help to define uniform criteria for LACM in the future.

## Conclusion

Studies specifically addressing LACM are extremely limited, and randomized controlled trials are lacking so far.

Our review represents an attempt to approach the diagnosis of LACM to define LACM more precisely by applying and combining several diagnostic criteria (Table 1 and Figure 8). These criteria reflect the electrical, structural, and mechanical remodeling of the atria and can be obtained in clinical practice. The interrelationship of the diagnostic methods and the derived parameters is interesting and important regarding the diagnosis of LACM and provides the opportunity for improved assessment.

Further investigation is urgently needed to improve the diagnostic capabilities and management of patients with LACM.

## Author Contributions

FK and MG contributed to conception and design of the review. FK wrote the first draft of the manuscript. MG wrote sections of the manuscript. Both authors contributed to manuscript revision, read, and approved the submitted version.

## Conflict of Interest

The authors declare that the research was conducted in the absence of any commercial or financial relationships that could be construed as a potential conflict of interest.

## Publisher’s Note

All claims expressed in this article are solely those of the authors and do not necessarily represent those of their affiliated organizations, or those of the publisher, the editors and the reviewers. Any product that may be evaluated in this article, or claim that may be made by its manufacturer, is not guaranteed or endorsed by the publisher.

## Abbreviations

LACM: left atrial cardiomyopathy
AF: atrial fibrillation
APW: amplified P-wave
BMI: body mass index
CT: computed tomography
ECG: electrocardiography
f-wave: fibrillatory wave
HFpEF: heart failure with preserved ejection fraction
LA: left atrium/left atrial
MRI: magnetic resonance imaging
PA-TDI: duration total atrial conduction time assessed by tissue doppler imaging
PET-CT: positron emission tomography – computed tomography
PTFV1: P-wave terminal force in lead V1
PVI: pulmonary vein isolation.
